# A slight recovery of soils from Acid Rain over the last three decades is not reflected in the macro nutrition of beech (*Fagus sylvatica*) at 97 forest stands of the Vienna Woods✰

**DOI:** 10.1016/j.envpol.2016.06.024

**Published:** 2016-06-22

**Authors:** Torsten W. Berger, Selina Türtscher, Pétra Berger, Leopold Lindebner

**Affiliations:** Department of Forest- and Soil Sciences, Institute of Forest Ecology, University of Natural Resources and Live Sciences (BOKU), Peter Jordan-Straße 82, 1190 Vienna, Austria

**Keywords:** *Fagus sylvatica*, Long-term trend, Plant nutrition, Soil acidification, Stemflow

## Abstract

Rigorous studies of recovery from soil acidification are rare. Hence, we resampled 97 old-growth beech stands in the Vienna Woods. This study exploits an extensive data set of soil (infiltration zone of stemflow and between trees area at different soil depths) and foliar chemistry from three decades ago. It was hypothesized that declining acidic deposition is reflected in soil and foliar chemistry. Top soil pH within the stemflow area increased significantly by 0.6 units in both H_2_O and KCl extracts from 1984 to 2012. Exchangeable Ca and Mg increased markedly in the stemflow area and to a lower extent in the top soil of the between trees area. Trends of declining base cations in the lower top soil were probably caused by mobilization of organic S and associated leaching with high amounts of sulfate. Contents of C, N and S decreased markedly in the stemflow area from 1984 to 2012, suggesting that mineralization rates of organic matter increased due to more favorable soil conditions. It is concluded that the top soil will continue to recover from acidic deposition. However, in the between trees areas and especially in deeper soil horizons recovery may be highly delayed. The beech trees of the Vienna Woods showed no sign of recovery from acidification although S deposition levels decreased. Release of historic S even increased foliar S contents. Base cation levels in the foliage declined but are still adequate for beech trees. Increasing N/nutrient ratios over time were considered not the result of marginally higher N foliar contents in 2012 but of diminishing nutrient uptake due to the decrease in ion concentration in soil solution. The mean foliar N/P ratio already increased to the alarming value of 31. Further nutritional imbalances will predispose trees to vitality loss.

## 1 Introduction

Reversibility of acidification is an extremely important topic, for both political and scientific reasons. From the beginning of the twentieth century, sulfur (S) emissions and deposition increased steadily and S deposition in forested ecosystems of north and central Europe peaked in the early 1980s reaching loads of more than 100 kg S ha^−1^ yr^−1^ (Prechtel et al., 2001). As a transboundary pollution issue, legislation to reduce acidifying emissions has taken place at an international level, through the Convention on Long-Range Transboundary Air Pollution of the United Nations Economic Commission for Europe (UN-ECE). E.g., in Austria, SO_2_ emissions declined from 1980 (385.000 t) to 2013 (17.000 t) by 95% (Umweltbundesamt, 2002, 2015). The topic is politically important because billions have been invested in cleaning up the emissions that cause Acid Rain, so it is worthwhile to know how much improvement has been achieved. Acidification research has moved down the agenda, however, the topic is also scientifically important because acid precursor emissions, and their subsequent removal, represent a profound large-scale perturbation to biogeochemical cycles (Likens et al., 1996). Unfortunately, the issue of recovery is still hardly debated (Lawrence et al., 2015). Revisiting the Acid Rain topic is worthy, since in many regions mass balance estimates of S are negative due to release of previously stored S, delaying the recovery of pH of soils and surface waters, depending on soil properties (e.g., see review by Watmough et al., 2005; references therein; Likens et al., 2002, 2013).

Stemflow of beech (*Fagus sylvatica*) represents a high input of water and elements, which is why deposition of acidifying substances may be significantly higher close to the stem compared to areas affected by throughfall only (e.g., Chang and Matzner, 2000; Kazda and Glatzel, 1984; Kazda et al., 1986; Matschonat and Falkengren-Grerup, 2000). As a consequence enhanced soil acidification around beech stems was observed, e.g., in the Solling area (Germany; Koch and Matzner, 1993) and in the Vienna Woods (Austria; Kazda, 1983; Rampazzo and Blum, 1992; Sonderegger, 1981).

Comparison between chemical parameters of soil from the infiltration zone of stemflow near the base of the stem and from the between trees area in 152 old-growth beech stands by Lindebner (1990; sample collection in 1984) in the Vienna Woods proved a significant impact of deposition of atmospheric pollutants: soil acidification, increased total sulfur contents and loss of base cations, especially in the infiltration zone. Focusing on the spatial heterogeneity of soil chemistry related to the distance from beech stems enables the study of recovery of differently polluted soil within the same stand, since the infiltration zone of beech stemflow received much higher S loads than the reference area between the trees in the past. Assuming that increasing soil solution fluxes with decreasing distance from the stem cause a quicker steady state of soil sulfate pools in response to currently decreasing inputs, Berger and Muras (2015) hypothesized that soil recovery from acidification will be hastened within the infiltration zone but may be delayed in the between trees areas.

Relations between soil nutrient availability and forest nutrition are poorly understood (Jandl et al., 2004; Miller and Watmough, 2009; van der Heijden et al., 2013). Foliar nutrient contents of the main European tree species (including *F. sylvatica*) declined in 20 of 22 significant cases (out of 6 tree species × 6 macronutrients = 36 cases) during 1992–2009 on the intensive forest monitoring plots of the ICP Forest Level II Programme, which were explained by the decrease in ion soil solution concentration (Jonard et al., 2015). Duquesnay et al. (2000) resampled 118 *F. sylvatica* stands in North-Eastern France and observed an increase in foliar N content and a decrease in foliar P, Ca and Mg contents between 1969–71 and 1996–97. In general, long-term foliar mineral changes for deciduous species and their relation to soil chemical data are scarce and are more common since the early 1990s (e.g., ICP Forest Level II network).

Rigorous studies of recovery from soil acidification are rare. Hence, we resampled 97 of 152 old-growth beech stands in the Vienna Woods, documented by Lindebner (1990) in the early 1980s. This study exploits an extensive data set of soil (infiltration zone of stemflow and between trees area) and foliar chemistry from three decades ago, and thus represents an opportunity that may be unique worldwide. We hypothesized that declining acidic deposition is reflected in soil and foliar chemistry and have developed our hypothesis into two specific research questions:

1) Have soils continued to acidify, stabilized or begun to recover as acidic deposition levels have declined?
2) Are changes of foliar nutrient contents over the last 3 decades reflected by corresponding patterns of soil available nutrients?

## 2 Materials and methods

### 2.1 Study area and study sites

The study area is spread throughout the Vienna Woods, a forested highland that forms the northeastern foothills of the Northern Limestone Alps, situated north, west and south of the City of Vienna (Austria). The bedrock of the major part of the Vienna Woods is Flysch. The Flysch zone is a narrow strip in the foothills of the Northern Limestone Alps from west to east throughout Austria, which is enlarged in the Viennese basin. Flysch consists mainly of old tertiary and mesozoic sandstones and clayey marls. The bedrock of the much smaller, southern part of the Vienna Woods is limestone, not covered by Flysch sediments. The total area is about 125.000 ha and is mainly forested land. Elevations range from about 180 m to over 800 m a.s.l. The mean annual precipitation varies between 600 and 900 mm, the mean annual temperature is 8–9 °C, and the two main wind directions are west (all over the year) and south-east (especially in fall and winter). The dominant tree species in the Vienna Woods is beech (*Fagus sylvatica*), representing 50% of the standing timber volume. Other species like oak (*Quercus* sp.), black pine (*Pinus nigra*) and Norway spruce (*Picea abie*s) make up a relatively small percentage of the forest cover (Plöchinger and Prey, 1974; Rieder, 2002).

**Fig. 1:**
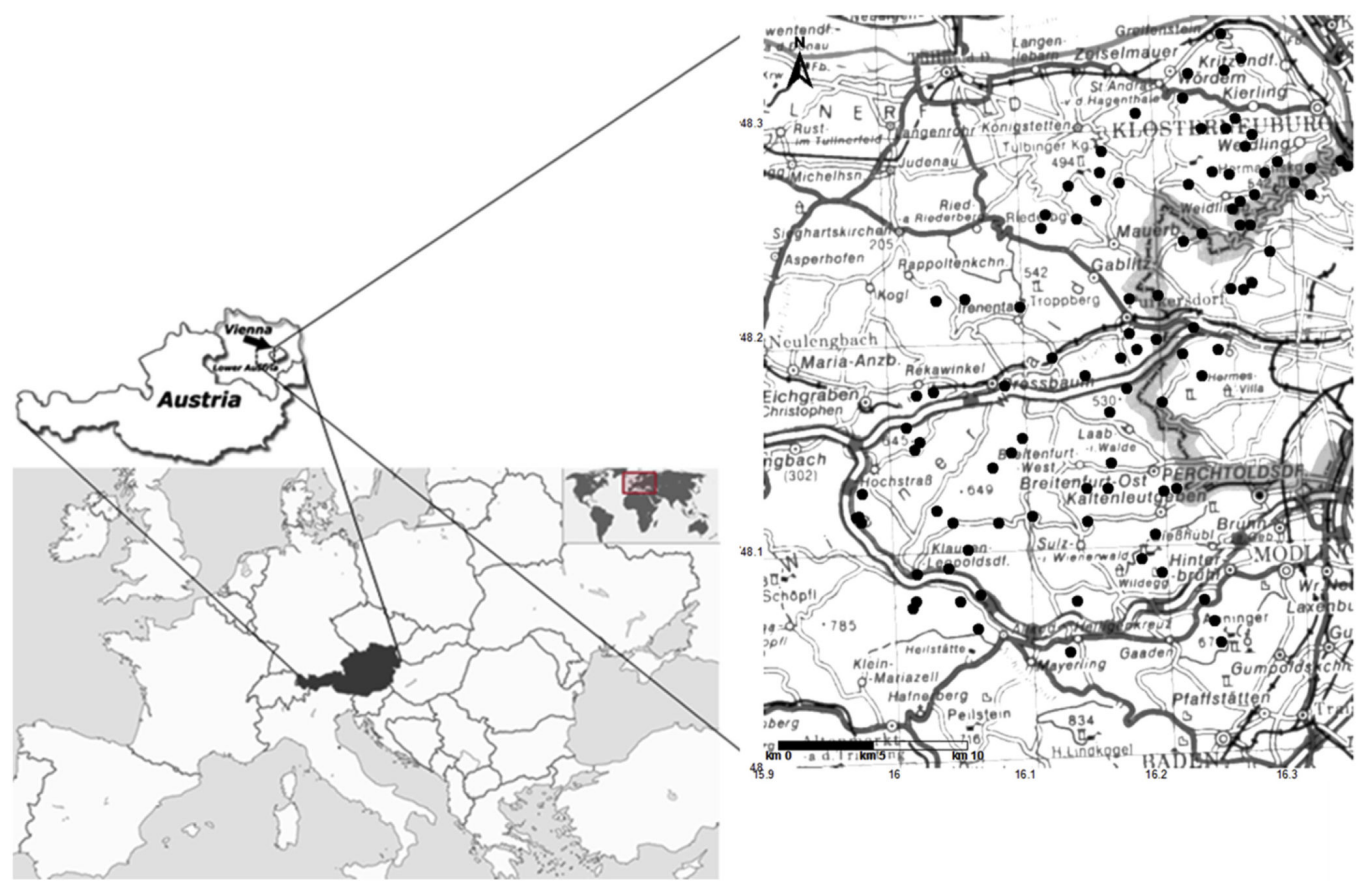
Location of 97 study sites in the Vienna Woods in 2012.

152 pure old-growth beech stands were selected in the early 1980s. At that time, all stands were older than 80 years and had a stand density index of ≥ 0.8. More details about the sites are given by Lindebner (1990). Three decades later, 97 of the 152 beech stands still existed for repeated sampling (Fig. 1). The rest of the sites had been cut. All soils on Flysch were classified as pseudogley (Scheffer and Schachtschabel, 2010; WRB classification: endostagnic cambisol), since horizons with a high fraction of fine material (loam to clay) cause temporary waterlogging (stagnation zone at approximately 40–50 cm soil depth). Soils on limestone at only 8 out of all 97 sites were classified as Kalkbraunlehm (Scheffer and Schachtschabel, 2010; WRB classification: endoleptic cambisol) and kept within the study because of similar soil texture and chemical properties. Nutrient release of both bedrocks is high and consequently the prevalent humus forms are mull to intermediate types between mull and moder, indicating quick turnover of the forest litter layer (usually less than 2 cm thickness) and nutrient rich soils.

### 2.2 Soil sampling and analysis

In summer 1984, seven top mineral soil samples (0–5 cm depth) per site from the infiltration zone of stemflow (20 cm downhill from the base of the stem, 1 sample per beech tree, S 0–5) and from the between trees area (at least 3 m away from beech stems, B 0–5) were taken with a cylinder (diameter 50 mm; height 50 mm). In each case, all 7 replicated samples per site were pooled before chemical analysis. One year later, in summer 1985, soil samples were taken with a soil auger (diameter 20 mm, half open steel pipe) from 30 to 40 cm (B 30–40) and 80–90 cm (B 80–90) soil depth in the between trees area (4 replications per site were pooled). Assuming that the deep soil parameters did not change within one year, we refer all these samples to the year 1984 throughout this paper.

In spring 2012, we repeated exactly the same sampling method as in 1984. In addition, we used the 20 mm diameter auger for sampling soil in 10–20 cm soil depth from the infiltration zone of stemflow (S 10–20, 7 samples adjacent to the 0–5 cm collections were pooled) and from the between trees area (B 10–20, the identical 4 soil profiles were used for B 10–20, B 30–40 and B 80–90).

Soil chemical parameters were determined by routine procedures as suggested by Blum et al. (1989) for the standardization of Austrian soil surveys: In 1984, mineral soil (< 2 mm) was analyzed for total content of C (Wösthoff Carmhomat ADG 8, Germany) and S (LECO SC 132, USA) according to ÖNORM L1080 and total N (Kjeldahl method, 2300 Kjeltec Analyzer Unit, Tecator, Sweden) according to ÖNORM L1082. In 2012, total C, S and N were analyzed by LECO SC 444 (USA, ÖNORM L1080). All methods were run parallel for several years, yielding comparable measurements. For the 1984 samples, no differentiation between organic and inorganic C was performed. However, for the 2012 samples, organic C was calculated total C minus C_CaCO3_ (Scheibler method: reaction of carbonates with HCl and volumetric determination of emerging CO_2_ according to ÖNORM L1084). Inorganic C turned out to be relevant only for B 80–90. Hence, measured total C contents of the 1984 samples in 80–90 cm depth were not used for this study. However, for all other 1984 samples total C was considered organic C. Calcium, Mg and K were measured as exchangeable cations (1 M ammonium acetate extract at pH 7, ÖNORM L1086) by graphite furnace atomic absorption spectrometry (GF-AAS, Perkin Elmer 3030, USA) in the 1984 samples and by inductive coupled plasma optical emission spectrometry (ICP-OES, Optima 3000 XL, Perkin Elmer, USA) in the 2012 samples. Again, both methods were run parallel for several years, yielding comparable results. Soil acidity was measured as pH with a glass Ag/AgCl combination electrode with KCl reference electrode: 10 g soil was mixed with 25 ml of 0.1 M KCl or deionized H_2_O, stirred, and the pH was measured next morning 30 min after stirring again (ÖNORM L1083).

### 2.3 Foliar sampling and analysis

In late August/early September 1984 and in early September 2012 leaf samples of beech were collected with a shot gun from the upper crown of two to three trees per site. All subsamples per site were pooled before analysis, yielding approximately 60–100 leaves.

Foliage samples were dried at 105 °C and ground. Total contents of C, N and S were analyzed as described for the soil samples above for the 1984 and 2012 samples. Phosphorus, Ca, Mg and K were measured as total contents after digestion with HNO_3_/HClO_4_ (ÖNORM L1085) by GF-AAS (1984 samples) and ICP-OES (2012 samples), respectively.

### 2.4 Data evaluation and statistics

Paired sample t-tests were performed to test the significance of different chemical parameters of the soil horizons (S 0–5, B 0–5, B 30–40 and B 80–90) and the foliage between the years 1984 and 2012. A repeated measures ANOVA was performed for each chemical soil parameter for each year separately (one way ANOVA, grouping factor: soil horizon) and results of multiple Bonferroni corrected paired comparison tests between the soil horizons were given. For the 1984 and 2012 data 4 horizons were compared with each other, while 6 soil horizons (S 0–5, S 10–20, B 0–5, B 10–20, B 30–40 and B 80–90) for the so-called 2012 extended data were used to evaluate whether recovery/acidification moved down the soil profile. Bivariate linear correlations were performed between total foliar nutrient contents and soil parameters for the years 1984 and 2012. All statistics were performed with the package IBM SPSS Statistics 21 software (IBM Corporation, Armonk, NY, US).

## 3 Results

### 3.1 Soil chemistry

Mean soil pH, contents of C_org_, N_tot_ and S_tot_ (mg g^−1^) and of exchangeable Ca, Mg and K (mg g^−1^) in the infiltration zone of stemflow near the base of the stem (S 0–5 and S 10–20) and in the between trees area (B 0–5, B 10–20, B 30–40 and B 80–90; given ranges are soil depths in cm) at the 97 beech stands of the Vienna Woods in 1984 and 2012 are given in Table 1.

Soil pH within the stemflow area (S 0–5) increased significantly by 0.6 units in both H_2_O- and KCl extracts from 1984 to 2012 (p < 0.001; as indicated by paired sample *t*-test). Soil pH (H_2_O) increased by 0.2 units at B 0–5 and there was a trend of slightly higher 2012 values in the deeper soil of the between trees area. Soil pH (KCl) increased from the top soil to the deep soil in the between trees area due to weathering release of base cations (as indicated by different letters in Table 1) indicating (not significant) trends of lower values in 2012 than 1984. Despite distinct signs of soil pH recovery in the stemflow area, present pHs were still lower than in the between trees area for comparable soil depths (see statistics in Table 1).

**Fig. 2:**
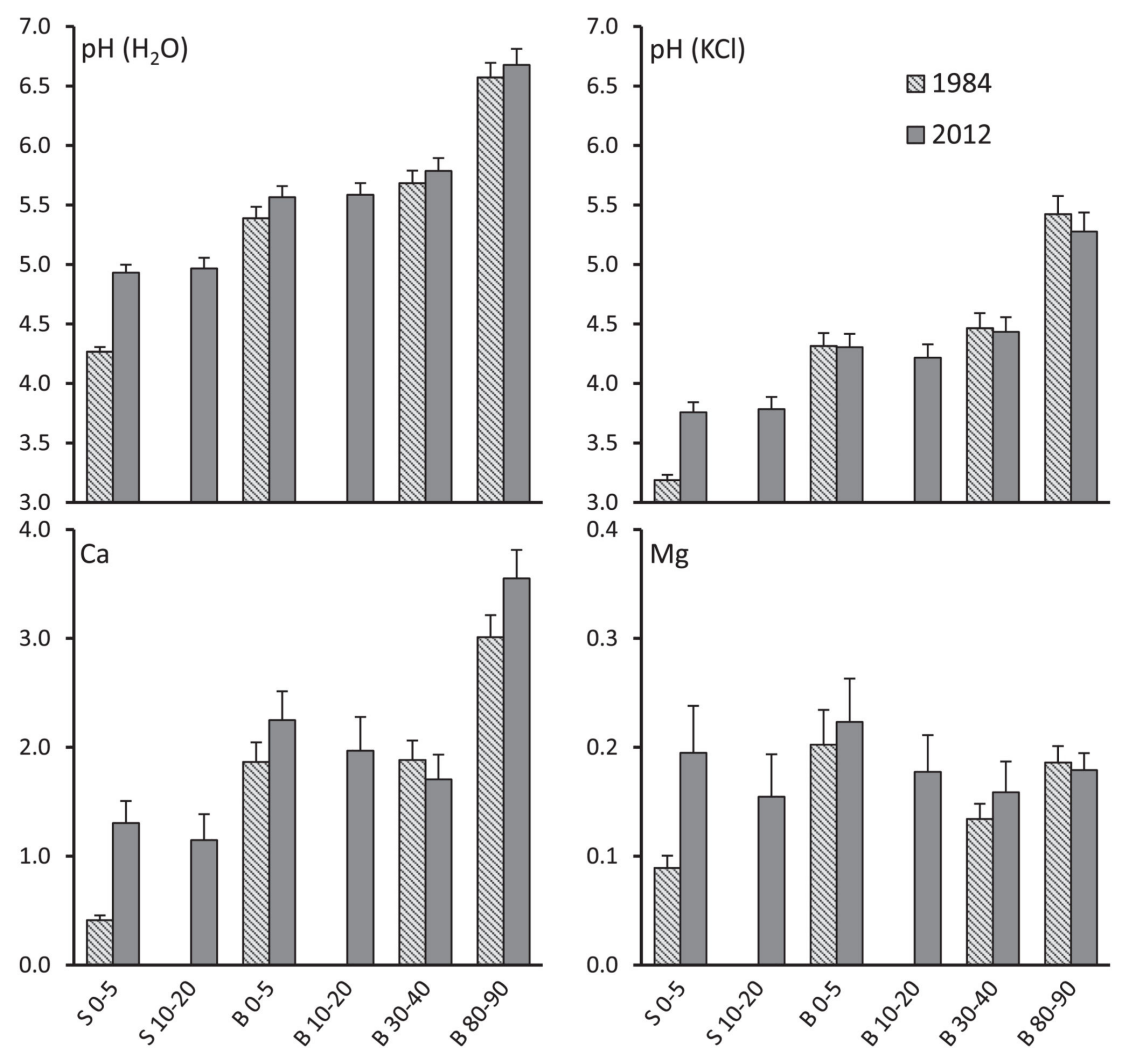
Mean soil pH (in H_2_O and KCl) and soil contents of Ca_exch_ and Mg_exch_ (mg g^−1^) in different soil depths of the stemflow area (S 0–5 and S 10–20) and the between trees area (B 0–5, B 10–20, B 30–40 and B 80–90) at 97 beech stands in 1984 and 2012. Error bars are ± S.E.

Exchangeable Ca increased markedly in the stemflow area and to a lower extent at B 0–5 and B 80–90 from 1984 to 2012. However, at B 30–40 Ca tended to be lower in 2012 than in 1984. The 2012 extended Ca values (between trees area) declined from 0–5 over 10–20 to 30–40 cm and increased thereafter in 80–90 cm depth (see Table 1 and Fig. 2). Exchangeable Mg showed similar patterns as Ca with significantly higher contents in 2012 than in 1984 in the stemflow area. In general, pH (H_2_O), pH (KCl), Ca and Mg showed similar gradients as plotted in Fig. 2.

**Fig. 3:**
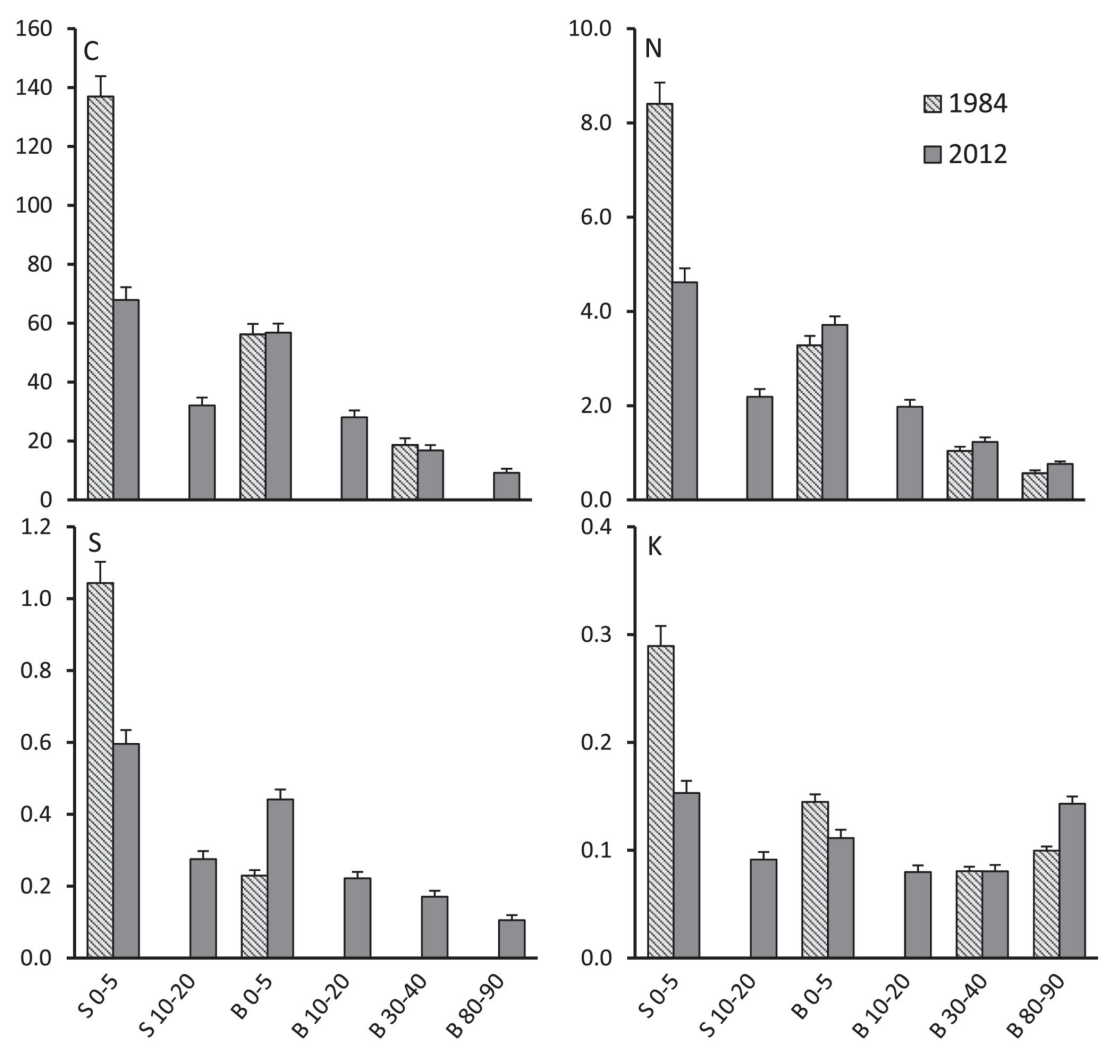
Mean soil contents of C_org_, N_tot_, S_tot_ and K_exch_ content (mg g^−1^) in different soil depths of the stemflow area (S 0–5 and S 10–20) and the between trees area (B 0–5, B 10–20, B 30–40 and B 80–90) at 97 beech stands in 1984 and 2012. Error bars are ± S.E.

Exchangeable K showed very contrasting patterns to Ca and Mg, respectively. From 1984 to 2012 K declined in the top soil (0–5 cm), especially in the stemflow area, and increased slightly at B 80–90. It is striking that general patterns of K, C, N and S were similar as visible in Fig. 3. Hence, unlike Ca and Mg, potassium was mainly associated with the soil organic matter. Contents of C, N and S decreased markedly (p < 0.001) in the stemflow area (S 0–5) from 1984 to 2012. The associated release of K from the top soil over the last 3 decades may have caused higher K contents in the deep mineral soil (B 80–90).

However recent contents of C, N and S were still higher in the stemflow area (S 0–5) than in the between trees area (B 0–5; as indicated by different letters in Table 1). Nitrogen contents in the soil profile of the between trees areas were marginally but significantly higher in 2012 than in 1984, reflecting long term effects of hardly changing N deposition in Austria since 1980 (Umweltbundesamt, 2002, 2015). Though S deposition declined markedly from 1984 to 2012 (Umweltbundesamt, 2002, 2015), S contents at B 0–5 were higher in 2012 than in 1984. Unfortunately, no soil S data were available for B 30–40 and B 80–90 from the year 1984.

### 3.2 Foliar chemistry

**Fig. 4:**
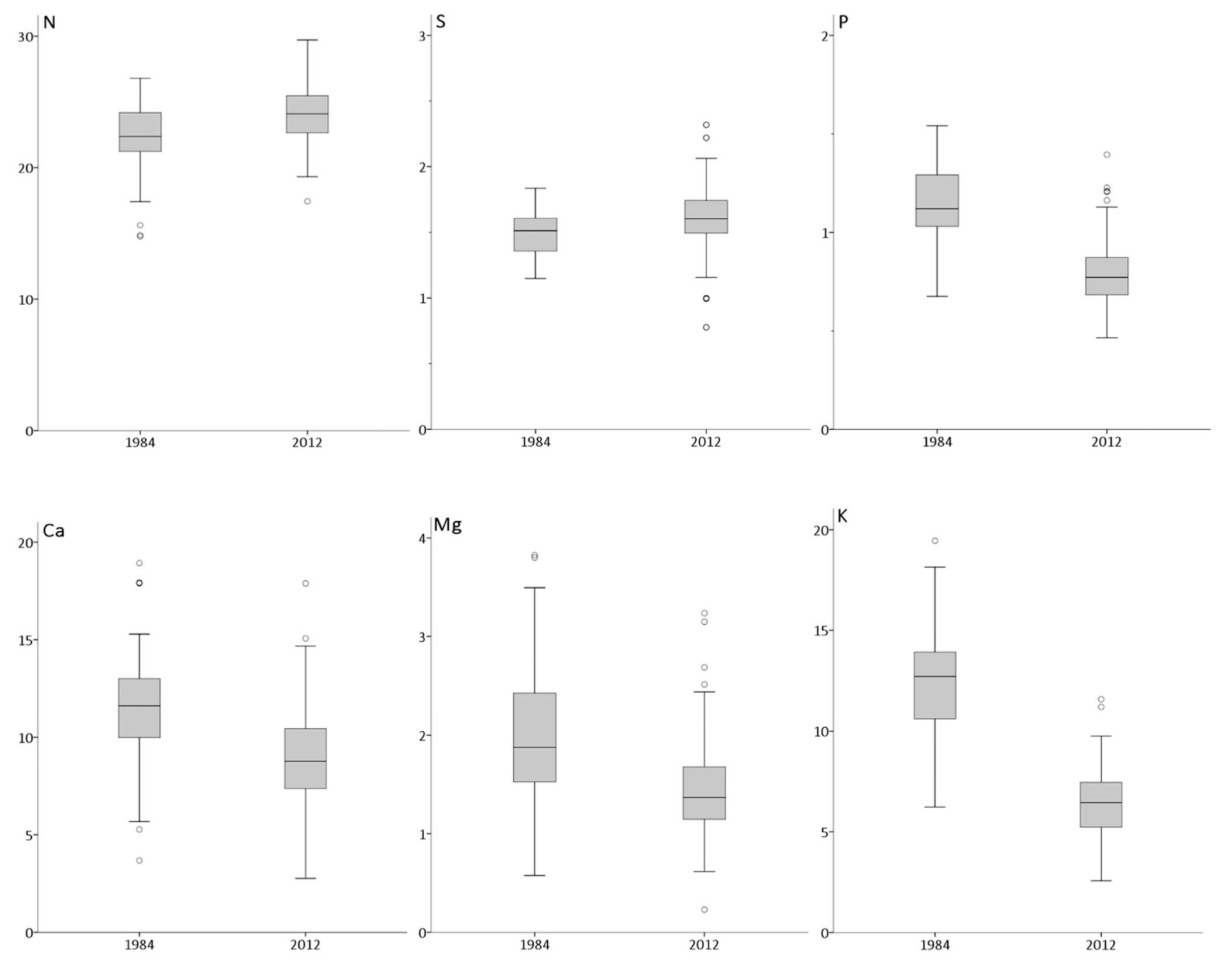
Box-and-whisker-plots illustrating total foliar contents of N, S, P, Ca, Mg and K (mg g^−1^) at 97 beech stands in the Vienna Woods. An individual plot may show 25%–75% (box; interquartile range, IQR), median (vertical line), whiskers (error bars; <1.5 IQR from upper or lower quartile) and outliers (circles; 1.5–3.0 IQR from upper or lower quartile). Extreme cases (>3.0 IQR from upper or lower quartile) are not shown.

**Fig. 5:**
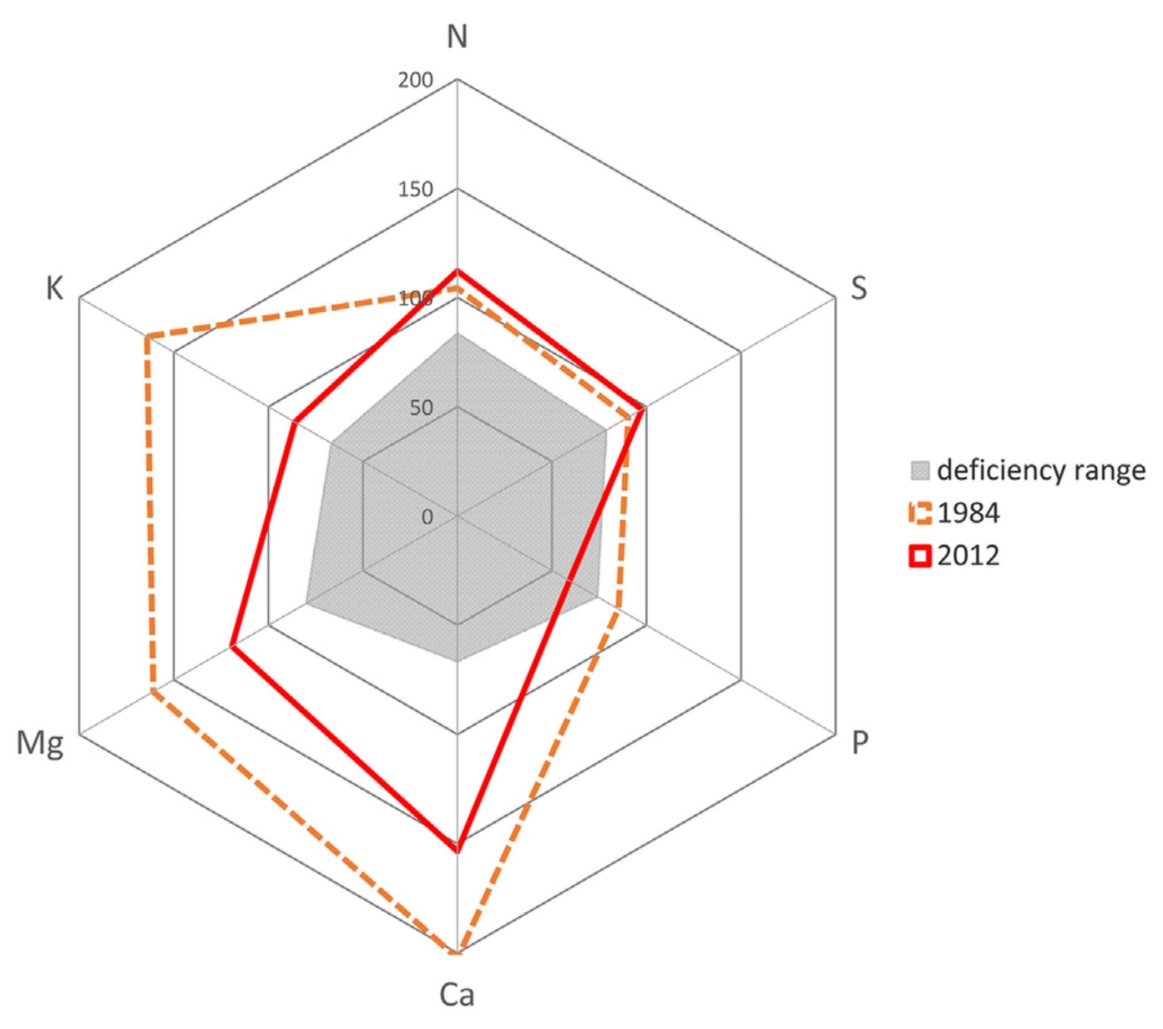
Mean macro nutrient contents of fresh beech foliage expressed as percentage of optimum nutrition (mean value of class range 2 according to Stefan et al., 1997): N: 21.50; S: 1.65; P: 1.35; Ca: 6.00; Mg: 1.25; K: 7.50; data in mg g^−1^) in 1984 and 2012. Grey area: deficiency range (upper value of class 1: N: 18.0; S: 1.3; P: 1.0; Ca: 4.0; Mg: 1.0; K: 5.0; data in mg g^−1^).

Foliar nutrition of the studied beech stands changed significantly for each of the 6 macro nutrients between 1984 and 2012. While contents of N and S increased, contents of P and the base cations Ca, Mg and K decreased within the last 3 decades (Table 2). Though min–max ranges were generally high (Table 2), it is striking that the variation (see interquartile ranges of box lengths and whiskers, plotted in Fig. 4) of foliar P, Mg and K contents over all 97 sites were declining as well from 1984 to 2012, strengthening the observed deterioration of tree nutrition for these elements. Nevertheless, only mean P nutrition switched from a lower optimum range (1984; class 2) into the deficient range (2012; class 1) according to Stefan et al. (1997; Fig. 5). The applied classification system of macro nutrient contents and nutrient ratios according to Stefan et al. (1997) is briefly summarized in the legend of Table 3 and results are given for all 97 beech stands. E.g., in 1984, 86% of the sites were allocated in P class 2, but in 2012, 85% were classified to P class 1. Though the highest decline of foliar nutrition was recorded for K (48%, from 1984 to 2012, expressed in mg g^−1^), mean K nutrition was still classified in the lower optimum range in 2012. In 2012, all other mean macro nutrient contents were still within the optimum (N, S) to lower surplus range (Mg, Ca) as well.

Higher foliar N but lower foliar contents of P, Ca, Mg and K in 2012 than in 1984 caused significant increases of the N/P, N/Ca, N/Mg and N/K ratios over time (Table 2). The mean N/S ratio did not change due to a simultaneous increase of S foliar contents from 1984 to 2012. However, all mean N/element ratios were still in the so-called harmonious range (class 2) according to Stefan et al. (1997), except the mean N/P ratio switched to the critical upper range (class 3, Table 3). Mineral nutrition of the base cations Ca, Mg and K seemed to be well balanced in 2012, though there was a preferential decline of foliar K over Ca and Mg within the last 3 decades.

**Fig. 6:**
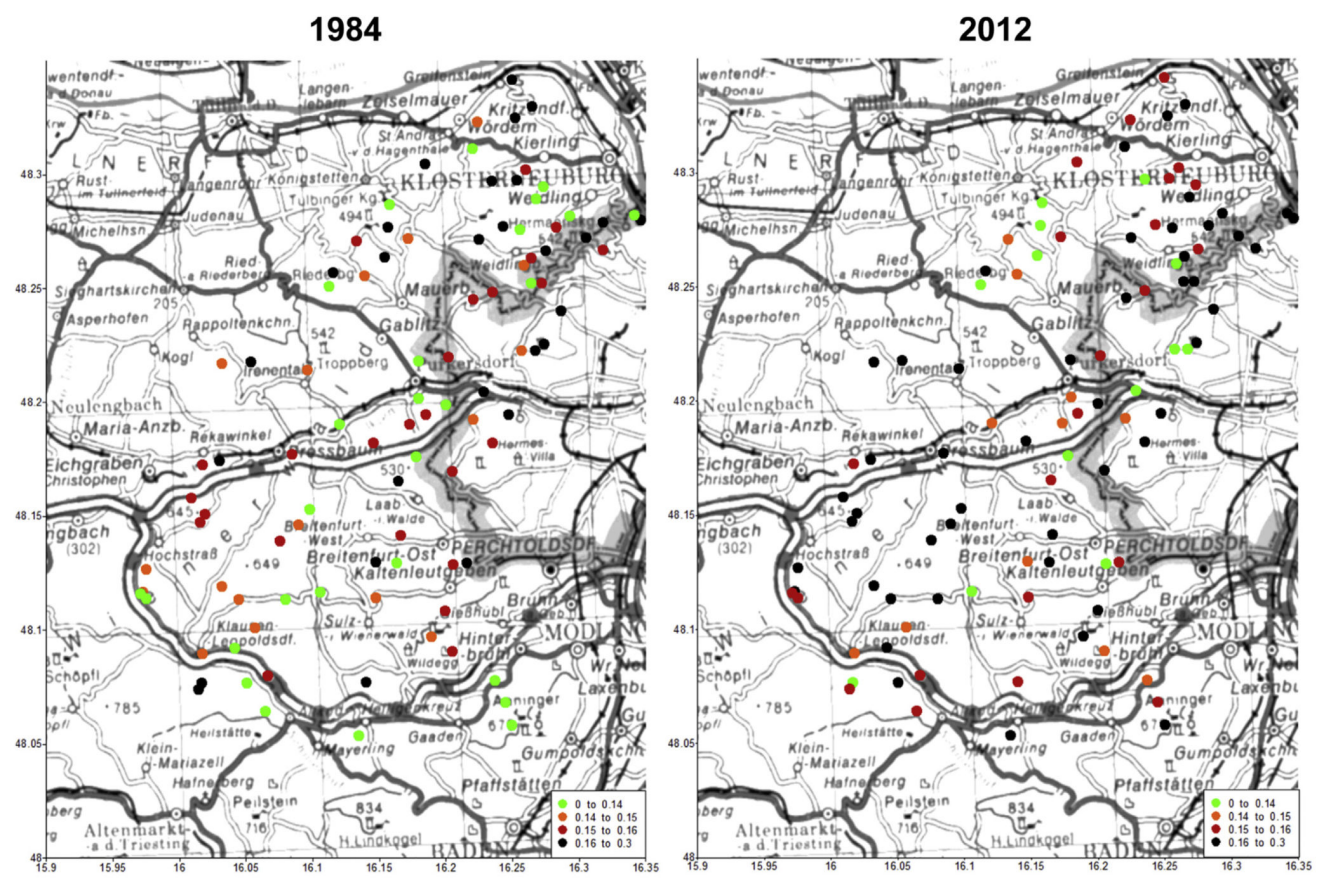
Geographical distribution of foliar S contents (sorted by classes) in 97 beech stands in the Vienna Woods in 1984 (left) and 2012 (right).

The geographical distribution of foliar S contents (sorted by classes) throughout the whole study area of the Vienna Woods is plotted in Fig. 6 for the years 1984 and 2012. Although it is difficult to detect clear spatial patterns, higher foliar S contents were clustered in the north of the study area and within the boarder of the City Vienna in 1984. In 2012, high foliar S contents were spread throughout the whole study area including the woods west and south of the City Vienna.

## 4 Discussion

### 4.1 Have soils continued to acidify, stabilized or begun to recover as acidic deposition levels have declined?

The terms soil acidification and recovery from acidification refer to a complex set of processes and can not be quantitatively described by a single index. A useful concept in this regard is that of capacity and intensity factors. According to Reuss and Johnson (1986) capacity in terms of soil acidity refers to the storage of protons or Al^3+^ or to the storage of base cations on the ion-exchange complex or in weatherable minerals. Intensity refers to the concentration in solution at any one time or, in the case of H^+^, the solution pH. One of the main goals of this study was to find out, how long it takes for soil to recover from Acid Rain. According to Lawrence et al. (2015) acidification of soil by acidic deposition is conceptualized as the acid leaching of bases at a rate that exceeds soil inputs from weathering and atmospheric deposition. Large decreases in acidic deposition may therefore enable soils to restore pools of exchangeable bases reduced during the period of high leaching rates (scenario 1: full recovery is possible). However, any uptake and subsequent export of bases by biomass should be included in the budget calculation.

On the other hand, based upon an excellent comment of an anonymous reviewer of this research proposal, we would like to ask critically: given that these soils, like most soils which have adequate moisture to produce leaching, acidify naturally, why would one assume that they will ever recover? It must have been the case that weathering and other mitigating factors did not keep pace with natural acidification pressures in the era before air pollution. Thus, how can we expect that the rate of weathering will now exceed the rate of acidification, even though the acidification rate has been reduced? Hence, the implicit assumption that soils will recover in the capacity sense from acidification has to be challenged (scenario 2: full recovery in the capacity sense is not possible). They can certainly recover in the intensity aspect, since total ionic concentrations will decline.

Soil pH (H_2_O) increase in the top soil over longer periods (Table 2) is in accordance with studies in Austria (Jandl et al., 2012), the Czech Republic (Reininger et al., 2011) and North America (Lawrence et al., 2015). However, a good demonstration of scenario 2 is the fact that pH of the between trees area in H_2_O tended to be higher but in KCl lower in 2012 than in 1984 (Fig. 2) which was caused by intensity versus capacity effects of acidity. Since pH in KCl eliminates intensity effects by exchanging H^+^ and to some extent Al species (and subsequent hydrolysis) from the soil exchanger (capacity effect) these data suggest that soil acidification moved downwards.

Comparing historic and recent contents of exchangeable base cations enables studies on soil recovery from acidification in the capacity sense as well. Both Ca and Mg contents increased in the top soil (S 0–5, B 0–5) from 1984 to 2012. However, the decline from 0 to 5 to 10–20 cm (infiltration zone) and from 0–5 over 10–20 to 30–40 cm (between trees area) in 2012 (Table 1, 2012 extended; Fig. 2), respectively, was not consistent with the conceptual model of recovery that assumes replenishment of bases from weathering as cation leaching fluxes are reduced by decreases in deposition. We suggest that this pattern was caused by mobilization of historic S and associated leaching of base cations with high amounts of sulfate, released within the last three decades. Top soil mineralization of organic S is the major source of net ${\text{SO}}_{4}^{2-}$ output on Flysch and the mean 2-year (2005–2007) net SO_4_-S balance on comparable beech stands (between trees area) was estimated −2.0 kg S ha^−1^ yr^−1^ (Berger et al., 2009). Simultaneously, this trend may be intensified by the fact that the Ca pump of beech (Berger et al., 2006) will alkalize the surface soil, but it will acidify the subsoil (e.g., 30–40 cm) from which the Ca is drawn. Hence, we conclude that net recovery of the whole soil profile in the capacity sense was small in the between trees area but showed clear differences between top and deep soil.

Berger and Muras (2015) hypothesized that using the microspatial heterogeneity of soil columns downhill of a beech stem enables the study of reversibility of soil acidification as a function of historic acid loads (stem area received much higher deposition loads in the past than the between trees area) and time (a space-for-time substitution is expected, since increasing soil solution fluxes due to additional stemflow with decreasing distance from the stem cause a quicker steady state of soil sulfate pools in response to decreasing inputs). This hypothesis is supported by our data, since soil recovery (expressed as increase of soil pH and exchangeable Ca and Mg) was accelerated in the infiltration zone of stemflow compared to the between trees area. However, despite distinct signs of soil recovery in the stemflow area, present pHs and contents of exchangeable Ca and Mg were still lower than in the between trees area for comparable soil depths after 3 decades of decreased acidic deposition (Fig. 2, statistics are given in Table 1).

In accordance with scenario 2 it seems questionable that a full recovery will be achieved at all. However, in accordance with scenario 1 the much higher reduction of acid loads in the stemflow area compared to the between trees area caused a quicker replenishment of base cations. Berger and Muras (2015) estimated S inputs 215 and 15 kg S ha^−1^ yr^−1^ in the stemflow area (27 cm downhill of the stem) and between trees area (300 cm downhill of the stem), respectively, based upon measurements by Sonderegger (1984) for a beech stand at the northern border of the City of Vienna in 1983. Due to the filtering and funneling effect of the beech canopy, any reduction of SO_2_ emissions must have caused an absolutely much higher reduction of acidic deposition in the stemflow area than in the between trees area.

It must be pointed out that other mitigating factors than weathering may have caused increased inputs of bases into the stemflow area. Besides the base cation pump of beech (see above), canopy leaching and associated deposition of base cations via stemflow are considered important factors improving the base status of the soil within the stemflow area. Berger et al. (2008) concluded for a beech stand on Flysch, 50 km west of Vienna, that cation leaching via throughall and stemflow was mainly driven by organic anions and bicarbonate but insignificantly by a cation exchange reaction with H^+^ as acidic deposition levels have declined.

Significantly higher C_org_ contents in the stemflow area (S 0–5) than in the between trees area (B 0–5; Table 1) are in accordance with other reports on declining C_org_ contents with increasing distance to a beech stem and were related to differences in the soil moisture regime, the amount of litterfall, and pH inhibition of decomposition (Koch and Matzner, 1993; Matschonat and Falkengren-Grerup, 2000; van der Heijden et al., 2013). Contents of C, N and S decreased markedly in the stemflow area (S 0–5) from 1984 to 2012, suggesting that this elemental loss was caused by improved mineralization rates of organic matter due to more favorable soil conditions (e.g, increased soil pH). Released divalent cations, which were sequestered in the organic matter were retained in the top mineral soil, while the monovalent cation K was replaced from the soil exchanger between 1984 and 2012 (Fig. 3). According to Reuss and Johnson (1986) physiochemical relationships are such that increased solution concentrations will increase the proportion of cations of higher valence versus those of lower valence on the soil exchanger (i.e., Ca^2+^ and Mg^2+^ will increase relative to H^+^ and K^+^). Overall, mineralization of organic C, N and S within the stemflow area is considered another mitigating factor, which accelerated soil pH recovery in the top soil of the stemflow area.

Though S deposition declined sharply from 1984 to 2012 (Umweltbundesamt, 2002, 2015), S contents at B 0–5 were higher in 2012 than in 1984, suggesting that presently a major part of historically deposited sulfate is cycling via plant uptake and litter fall through the organic S pool (e.g., Alewell, 2001). The fact that a considerable proportion of atmospherically deposited sulfate is cycled through the organic S pool before being released to soil solution and stream water is indicated by stable S isotopes (^34^S/^32^S ratios; e.g., Zhang et al., 1998; Alewell et al., 1999). Berger et al. (2009) concluded for comparable beech sites on Flysch that net mineralization of pedogenic S in the top soil is the major reason for negative ${\text{SO}}_{4}^{2-}$ input - output budgets. Hence, the release of this accumulated S in the between trees area may be a reason for the observed slow recovery of soil pH (KCl, Fig. 2) despite reductions in SO_4_ and H^+^ deposition.

Finally, we have to give a complex answer to our research question 1: Soils i) partly continued to acidify in deeper layers, ii) have stabilized or just slightly recovered in the top layers of the between trees area, and iii) recovered markedly in the top layers of the stemflow area without reaching as favorable soil conditions as in the between trees area.

### 4.2 Are changes of foliar nutrient contents over the last 3 decades reflected by corresponding patterns of soil available nutrients?

Mean foliar N content increased significantly from about 22 mg g^−1^ to 24 mg g^−1^ within the last three decades. This increase is relatively small and mathematically cannot be the cause of significantly increased N/P, N/Ca, N/Mg and N/K ratios within the last 3 decades, because the same elemental ratios would increase by dividing foliar N content of the year 1984 with element contents of the year 2012. Hence, as suggested by Jonard et al. (2015) it is possible that tree productivity increased resulting from high N deposition and from global increase in atmospheric CO_2_ leading to higher nutrient demand by trees. In case this high nutrient demand cannot be supplied via the plant available soil nutrient pool, foliar N/nutrient ratios will increase as recorded in our study and elsewhere in Europe (e.g., Duquesnay et al., 2000; Flückiger and Braun, 1998; Jonard et al., 2009a, 2012, 2015). Nitrogen fertilization experiments with potted young beech caused increased N/element ratios, and N/Mg and N/K ratios after addition of 200 kg N ha^−1^ were in the same range as reported by us for the year 2012 (Table 2), however, no information is given about the fertility of the experimental soil (Flückiger and Braun, 1998). The only given information is that these authors used a loamy sand, suggesting much lower total contents of exchangeable Ca and Mg than for the loamy to clayey soils of the Vienna Woods. According to a pot experiment with oak seedlings, N fertilization caused critical foliar N/element ratios on an acidic nutrient-poor soil but did not change the ratios on an alkaline nutrient-rich soil at all (Berger and Glatzel, 2001). The soils of the study area are nutrient rich as well and exchangeable soil Ca and Mg increased from 1984 to 2012. Nitrogen contents in the soil profile of the between trees areas were only marginally (though significantly) higher in 2012 than in 1984. Hence, we conclude that higher plant uptake of P, Ca, Mg and K in 1984 than in 2012 caused the observed increase of N/nutrient ratios over time.

In agreement with Jonard et al. (2012), we suggest that the decrease in ion concentration in the soil solution caused decreased uptake rates of base cations in 2012. According to the mobile anion concept by Reuss and Johnson (1986) “the concentration of anions in solution will control the total concentrations of cations, while the composition of cations in solution should be controlled by equilibration with what is usually a large pool of cations adsorbed on soil particles.” Since acidic deposition declined from 1984 to 2012, we assume that base cation concentrations in the soil solution of the studied soils, characterized by high base saturations, declined as well. On comparable sites on Flysch in Austria, Berger et al. (2004) measured significantly higher Ca and Mg concentrations in stem wood of spruce for the pure spruce-than for the mixed beech-spruce stands in spite of lower Ca and Mg stores in the soil. These authors assumed that acidification caused by pure spruce (which may be similar to acidification by Acid Rain) mobilized these cations temporarily, increasing soil solution contents and consequently stem wood concentrations. Hence, plant available soil nutrients (capacity parameter) are considered much poorer indicators of plant nutrition than actual elemental soil solution concentrations (intensity parameter). This context helps to understand why foliar Ca and Mg declined (Table 2) despite increasing exchangeable Ca and Mg soil pools (Table 1) from 1984 to 2012.

Undisturbed soil cores were taken only from the top soil (0–5 cm), however, no bulk densities were estimated for the 1984 samples. Assuming the same bulk densities for 1984 and 2012 an increase of Ca from 0.41 to 1.31 (S 0–5) and from 1.87 to 2.25 (B 0–5, Table 1) mg g^−1^ corresponds to an increase of total Ca pools from 15.7 to 45.1 (S 0–5) and from 76.6 to 90.7 (B 0–5) g m^−2^ for 0–5 cm soil depth, respectively. Similarly, the increase of Mg from 0.09 to 0.19 (S 0–5) and from 0.20 to 0.22 (B 0–5, Table 1) mg g^−1^ corresponds to an increase of total Mg pools from 3.3 to 6.2 (S 0–5) and from 7.5 to 8.1 (B 0–5) g m^−2^ for 0–5 cm soil depth, respectively. Though the estimated net-increases of plant available Ca and Mg pools in 0–5 cm soil depth were roughly in the order of element stocks in fresh foliage plus twigs of a mature beech stand (Jochheim et al., 2007), the detected changes seem not relevant for nutrition of beech as pointed out above (Fig. 5).

Pearson correlation coefficients between foliar nutrient contents and soil parameters in the between trees area (B 0–5) are given in Table 4 for the years 1984 and 2012. It is striking that, except for S, these relations did not change between the years, though soil nutrient pools did change. This finding supports the theory of unclear relations between soil chemical fertility and plant response. Though Ca and Mg foliar contents were significantly correlated with soil chemical parameters within the year of observation, the highest *R* of 0.69 means that only 48% (*R^2^*) of the variation of the nutrient foliar content can be explained by the corresponding soil nutrient content.

Potassium was deleted from the soil exchanger in the top soil between 1984 and 2012 (Table 1) and this pattern was reflected in K foliar contents as well (Table 2). However, in the year of observation, plant available soil K was not related to foliar K content at all (Table 4). Negative correlations between soil nutrients (especially Mg) and foliar K indicate that K/nutrient antagonisms in the soil or in the plant may play an important role for K nutrition. We suggest that the observed surplus K nutrition in 1984 was caused by replacement of this monovalent cation from the soil exchanger during the peak of the Acid Rain period.

Increases of foliar S contents from 1984 to 2012 (Table 2) may be discussed controversially to the literature. On one hand, reduced SO_2_ emissions will cause reduced foliar S contents via the air path (Fürst et al., 2003; Jonard et al., 2012, 2015), on the other hand, our results match to numerous studies on present release of historic S via desorption of inorganic sulfate from the soil exchanger (mainly relevant for acidic soils) or mineralization of organic S (e.g., Likens et al., 2002, 2013; Watmough et al., 2005). The fact that total S soil content was positively related with foliar Ca- and Mg contents in 2012 but not in 1984 (Table 4) supports the hypothesis that presently mineralization of organic S releases sulfate into the soil solution that is accompanied by these cations (note: at three selected beech stands on Flysch in the Vienna Woods organic S amounted 72–89% of total S; data not shown). Our suggestion that S impact on foliar nutrition switched from the air path to the soil path is roughly concluded from Fig. 6. In 1984, high foliar S contents were clustered in the north of the study area and within the boarder of the City Vienna in 1984, because the prevailing wind direction during high-pressure episodes is from south-east to north-west, suggesting that local SO_2_ emissions of the city affected foliar S nutrition in 1984. In 2012, high foliar S contents were spread throughout the whole study area including the woods west and south of the City Vienna, indicating a transition from a local to a more regional S impact via the soil path.

Phosphorus nutrition declined significantly and the mean N/P ratio increased to the alarming value of 31 from 1984 to 2012, indicating a severe nutritional imbalance (Table 2, Fig. 5). In 2012, 86% of the 97 sites were assigned to class 3 (Table 3). Foliar P contents were not related to measured soil parameters, except in 1 out of 14 cases (Table 4; note that soil P contents were not measured). Our data do not give an explanation for the observed deterioration of P nutrition, but we suggest that i) lower soil pHs of the top soil in the 1980s preferred the dominant uptake of P as H_2_PO4^-^ according to Marschner (2002). ii) Large decrease in beech foliar P content could partly be explained by decrease in mycorrhizal abundance due to atmospheric N deposition (Duquesnay et al., 2000; compare slight increase of soil N contents in the between trees area in Fig. 3), because mycorrhizae play a major role in P uptake. iii) The reduction in the thickness of the forest floor which plays a major role in P nutrition (Jonard et al., 2009b) could be another reason in accordance to the observed loss of organic matter within the stemflow area (see patterns of soil C, N and S in Fig 3). iv) Since sulfate and phosphate are partly adsorbed on the same anion exchangers of the soil constituents (Geelhoed et al., 1997), sulfate via acidic deposition in the 1980s and thereafter via release from the organic soil (as discussed above) could have depleted plant available phosphate as suggested by Jonard et al. (2015).

Upward trend in foliar mass (dilution effect) and increase of a stand’s productivity (storage of nutrients in stems and branches) may cause declining foliar nutrient contents as well. In Germany, comparison between an 80 and 122 years old beech stand revealed decreases in foliar N, P and Mg contents with age by −7, −14 and −7% but no clear trend for Ca and K (Cole and Rapp, 1981). In our study, foliar dilution effects are considered negligible since foliar N contents increased and changes for P and Mg were much higher anyway. The periodic annual increment in volume of a forest stand increases to a maximum value as a tree matures and then declines during the rest of the silvicultural cycle. Since the selected beech stands were older than 80 years in 1984, the stands had passed the period of maximum growth already before the beginning of the study period (Jonard et al., 2015). In addition, increasing internal redistribution reduces the nutrient demands of old trees (Kimmins, 1997), justifying the assumption to neglect stand age effects on measured soil parameters in this study as well.

Finally, we have to give a complex answer to our research question 2 as well: i) In general, the plant available nutrient pool (capacity parameter) is a poor indicator for plant nutrition, because soil solution chemistry (intensity parameter) seems to control foliar chemistry. ii) Slight increases of total N and S and sharp decreases of exchangeable K soil contents in the between trees area were accompanied by concomitant foliar nutrient changes over time. iii) Despite an observed increase of soil Ca and Mg contents from 1984 to 2012 the corresponding foliar contents declined.

## 5 Conclusions

The beech stands of the Vienna Woods showed no sign of recovery from acidification although S deposition levels decreased. Release of historic S even increased foliar S contents. Base cation levels in the foliage declined but are still adequate for beech trees. The top soil will recover from acidic deposition, as already recorded in the infiltration zone of stemflow near the base of the stem. However, in the between trees areas and especially in deeper soil horizons recovery may be highly delayed. The unbalanced reduction in atmospheric deposition will reinforce N eutrophication of forest ecosystems and the disorders in tree nutrition. The mean foliar N/P ratio already increased to the alarming value of 31. Further nutritional imbalances will predispose trees to vitality loss.

## Figures and Tables

**Table 1:** Mean soil pH, mean contents of C_org_, N_tot_ and S_tot_ (mg g^−1^) and of exchangeable Ca, Mg and K (mg g^−1^) in the infiltration zone of stemflow near the base of the stem (S 0–5 and S 10–20) and in the between trees area (B 0–5, B 10–20, B 30–40 and B 80–90; given ranges are soil depths in cm) at 97 beech stands of the Vienna Woods in 1984 and 2012.

Parameter	S 0-5			S 10-20	B 0-5			B 10-20	B 30-40			B 80-90		
	1984		2012	2012	1984		2012	2012	1984		2012	1984		2012
*pH (H_2_O)*	4.3		4.9	5.0	5.4		5.6	5.6	5.7		5.8	6.6		6.7
paired *t*-test		***				**				ns			ns	
multiple comparison 1984	A				B				C			D		
multiple comparison 2012			a				b				c			d
multiple comparison 2012 extended			*a*	*a*			*b*	*b*			*c*			*d*
*pH (KCl)*	3.2		3.8	3.8	4.3		4.3	4.2	4.5		4.4	5.4		5.3
paired *t*-test		***				ns				ns			ns	
multiple comparison 1984	A				B				C			D		
multiple comparison 2012			a				b				b			c
multiple comparison 2012 extended			*a*	*a*			*bc*	*b*			*c*			*d*
*Ca_exch_*	0.41		1.31	1.15	1.87		2.25	1.97	1.89		1.71	3.01		3.55
paired *t*-test		***				*				ns			*	
multiple comparison 1984	A				B				B			C		
multiple comparison 2012			a				b				a			c
multiple comparison 2012 extended			*ab*	*a*			*d*	*cd*			*bc*			*e*
*Mg_exch_*	0.09		0.19	0.15	0.20		0.22	0.18	0.13		0.16	0.19		0.18
paired *t*-test		**				ns				ns			ns	
multiple comparison 1984	A				C				B			C		
multiple comparison 2012			ab				b				a			ab
multiple comparison 2012 extended			*bc*	*a*			*c*	*ab*			*ab*			*abc*
*C_org_*	136.94		67.89	32.07	56.23		56.77	28.07	18.65		16.82			9.17
paired *t*-test		***				ns				ns				
multiple comparison 1984	C				B				A					
multiple comparison 2012			d				c				b			a
multiple comparison 2012 extended			*e*	*c*			*d*	*c*			*b*			*a*
*N_tot_*	8.41		4.62	2.19	3.28		3.71	1.98	1.04		1.23	0.57		0.76
paired *t*-test		***				**				**			**	
multiple comparison 1984	D				C				B			A		
multiple comparison 2012			d				c				b			a
multiple comparison 2012 extended			*e*	*c*			*d*	*c*			*b*			*a*
*S_tot_*	1.04		0.60	0.28	0.23		0.44	0.22			0.17			0.11
paired *t*-test		***				***								
multiple comparison 1984	B				A									
multiple comparison 2012			b				a							
multiple comparison 2012 extended			*f*	*d*			*e*	*c*			*b*			*a*
*K_exch_*	0.29		0.15	0.09	0.14		0.11	0.08	0.08		0.08	0.10		0.14
paired *t*-test		***				***				ns			***	
multiple comparison 1984	D				C				A			B		
multiple comparison 2012			c				b				a			c
multiple comparison 2012 extended			*d*	*b*			*c*	*a*			*ab*			*d*

Paired sample t-tests were performed to test significance of differences between the years 1984 and 2012: ns: not significant;

* p ≤ 0.05;

** p ≤ 0.01;

*** p ≤ 0.001.

A repeated measures ANOVA was performed for each parameter and year separately and results of multiple Bonferroni corrected paired comparison tests between the soil horizons are given (different letters indicate significant differences, p < 0.05; A, a and *a* represent the lowest means of 1984, 2012 and 2012 extended, respectively).

**Table 2:** Mean element content (mg g^−1^) and nutrient ratios of fresh beech foliage in 1984 and 2012. The min-max range includes extreme cases, which are not shown in Fig. 4.

	N		S		P		Ca		Mg		K		Ca/Mg	
	1984	2012	1984	2012	1984	2012	1984	2012	1984	2012	1984	2012	1984	2012
min	14.76	17.43	1.15	0.78	0.68	0.47	3.68	2.76	0.58	0.23	6.24	2.56	3.20	2.90
max	26.80	29.71	1.84	2.32	1.54	1.39	30.21	24.48	5.52	3.79	19.45	11.58	16.50	16.00
**Mean**	**22.41**	**24.09**	**1.49**	**1.61**	**1.15**	**0.80**	**12.14**	** 9.20**	**2.01**	**1.49**	**12.31**	**6.44**	** 6.38**	** 6.71**

paired *t*-test	***		***		***		***		***		***		ns	
	N/S		N/P		N/Ca		N/Mg		N/K		K/Ca		K/Mg	
	1984	2012	1984	2012	1984	2012	1984	2012	1984	2012	1984	2012	1984	2012

min	8.90	10.20	11.00	18.10	0.60	1.00	3.50	6.00	1.00	2.00	0.40	0.20	1.20	0.80
max	20.20	32.50	34.20	45.50	6.10	7.80	40.00	93.80	3.80	10.10	3.80	2.70	26.50	32.40
**Mean**	**15.19**	**15.22**	**19.98**	**31.09**	**2.02**	**2.86**	**12.45**	**18.35**	**1.92**	** 4.02**	**1.10**	**0.77**	** 6.96**	** 5.10**

paired *t*-test	ns		***		***		***		***		***		***	

Paired sample *t*-tests were performed to test significance of differences between the years 1984 and 2012: ns: not signifiant; *p ≤ 0.05; **p ≤ 0.01;

*** p ≤ 0.001.

**Table 3:** Percentage of sites (*N* = 97), classified by macro nutrient contents (mg g^−1^) in (1) deficient-, (2) optimum- and (3) surplus range, as well as by corresponding nutrient ratios (class 1: lower range, class 2: medium/harmonious range, class 3: upper range) according to Stefan et al. (1997).

Class Limit	N			S			P			Ca			Mg			K			Ca/Mg		
	1	2	3	1	2	3	1	2	3	1	2	3	1	2	3	1	2	3	1	2	3
	18.0–25.0			1.3–2.0			1.0–1.7			4.0–8.0			1.0–1.5			5.0–10.0			3.7–8.0		

1984	3	80	17	11	89	0	13	86	1	1	5	94	2	20	78	1	16	83	3	81	16
2012	1	69	30	5	91	4	85	15	0	1	36	63	12	47	41	19	79	2	6	74	20
Class Limit	N/S			N/P			N/Ca			N/Mg			N/K			K/Ca			K/Mg		
	1	2	3	1	2	3	1	2	3	1	2	3	1	2	3	1	2	3	1	2	3
	9.0–19.2			10.6–25.0			2.3–6.3			12.0–25.0			1.8–5.0			0.6–2.5			3.3–10.0		

1984	1	97	2	0	93	7	73	26	1	48	50	2	39	60	1	5	94	1	6	88	6
2012	0	95	5	0	14	86	22	77	1	11	81	8	0	85	15	20	79	1	18	79	3

**Table 4:** Coefficients of correlation (Pearson) between total foliar nutrient contents and soil parameters in the between trees area in 0–5 cm soil depth (B 0–5) in 1984 and 2012.

			foliar content					
Parameter [mg g^-1^]			N	S	P	Ca	Mg	K
soil parameter (B 0-5)	pH (H_2_O)	1984	-0.26	-0.32	-0.14	0.60	0.55	-0.22
		2012	0.08	0.14	-0.02	0.67	0.46	-0.11
						
	pH (KCl)	1984	-0.24	-0.32	-0.13	0.60	0.59	-0.23
		2012	0.14	0.17	-0.02	0.68	0.51	-0.17
						
	N_tot_	1984	-0.24	-0.18	-0.18	0.32	0.40	-0.29
		2012	-0.02	0.15	-0.10	0.42	0.42	-0.22
						
	S_tot_	1984	0.02	0.01	-0.17	-0.08	-0.03	-0.11		> 0.05
		2012	-0.05	0.10	-0.13	0.49	0.41	-0.21		
						
	Ca_exch_	1984	-0.22	-0.28	-0.13	0.64	0.51	-0.17		< 0.05
		2012	0.15	0.15	-0.05	0.62	0.55	-0.26		
						
	Mg_exch_	1984	-0.14	-0.29	-0.29	0.26	0.62	-0.51		< 0.01
		2012	0.07	-0.01	-0.14	0.36	0.69	-0.40		
						
	K_exch_	1984	-0.18	-0.09	0.12	0.63	0.17	0.07		< 0.001
		2012	-0.00	0.18	0.16	0.69	0.08	0.12		
